# ICI efficacy information portal: a knowledgebase for responder prediction to immune checkpoint inhibitors

**DOI:** 10.1093/narcan/zcad012

**Published:** 2023-03-03

**Authors:** Jiamin Chen, Daniel Rebibo, Jianquan Cao, Simon Yat-Man Mok, Neel Patel, Po-Cheng Tseng, Zhenghao Zhang, Kevin Y Yip

**Affiliations:** Department of Computer Science and Engineering, The Chinese University of Hong Kong, Shatin, New Territories, Hong Kong SAR; Sanford Burnham Prebys Medical Discovery Institute, La Jolla, CA, USA; Department of Computer Science and Engineering, The Chinese University of Hong Kong, Shatin, New Territories, Hong Kong SAR; School of Biomedical Sciences, The Chinese University of Hong Kong, Shatin, New Territories, Hong Kong SAR; Department of Computer Science and Engineering, The Chinese University of Hong Kong, Shatin, New Territories, Hong Kong SAR; Department of Computer Science and Engineering, The Chinese University of Hong Kong, Shatin, New Territories, Hong Kong SAR; Department of Mathematics, Indian Institute of Technology, Hauz Khas, New Delhi, India; Department of Computer Science and Engineering, The Chinese University of Hong Kong, Shatin, New Territories, Hong Kong SAR; Department of Computer Science and Engineering, The Chinese University of Hong Kong, Shatin, New Territories, Hong Kong SAR; Department of Computer Science and Engineering, The Chinese University of Hong Kong, Shatin, New Territories, Hong Kong SAR; Sanford Burnham Prebys Medical Discovery Institute, La Jolla, CA, USA

## Abstract

Immune checkpoint inhibitors (ICIs) have led to durable responses in cancer patients, yet their efficacy varies significantly across cancer types and patients. To stratify patients based on their potential clinical benefits, there have been substantial research efforts in identifying biomarkers and computational models that can predict the efficacy of ICIs, and it has become difficult to keep track of all of them. It is also difficult to compare findings of different studies since they involve different cancer types, ICIs, and various other details. To make it easy to access the latest information about ICI efficacy, we have developed a knowledgebase and a corresponding web-based portal (https://iciefficacy.org/). Our knowledgebase systematically records information about latest publications related to ICI efficacy, predictors proposed, and datasets used to test them. All information recorded is checked carefully by a manual curation process. The web-based portal provides functions to browse, search, filter, and sort the information. Digests of method details are provided based on the original descriptions in the publications. Evaluation results of the effectiveness of the predictors reported in the publications are summarized for quick overviews. Overall, our resource provides centralized access to the burst of information produced by the vibrant research on ICI efficacy.

## Graphical Abstract

**Graphical Abstract:**
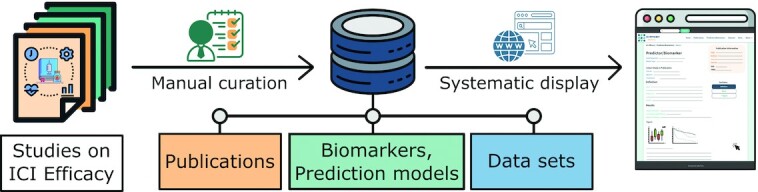
ICI efficacy information portal provides centralized access to latest research on efficacy of cancer immune checkpoint inhibition therapies, with a manually curated database of publications, biomarkers/prediction models, and datasets.

## INTRODUCTION

Immune checkpoint inhibition, also referred to as immune checkpoint blockade, is a type of cancer therapy that has led to durable responses in various cancer types, including cancers that are otherwise difficult to treat. Its main working principle is to block immunoregulatory ‘checkpoint’ molecules, thereby restoring the activities of immune cells against tumor cells (1–3). The most common ICI targets are cytotoxic T lymphocyte antigen 4 (CTLA-4) and programmed cell death 1 (PD-1), or its ligand (PD-L1). Inhibiting CTLA-4 can result in the expansion of CD4^+^ effector T cells, while inhibiting PD-1/PD-L1 can reinvigorate exhausted CD8^+^ T cells. A series of additional ICI targets have also been investigated, such as the T cell-expressed inhibitory receptors LAG-3, TIM-3 and TIGIT, and inhibitory ligands in the B7 family (4–6).

ICI has been highly effective in some cancer types. For example, in an early study of the PD-1 inhibitor Nivolumab (7), among the 23 patients with Hodgkin's lymphoma receiving the treatment, an objective response was reported in 20 patients (87% of the cohort), including four patients with complete response, defined as having substantial tumor regression. Even for the three patients not achieving an objective response, a stable disease was maintained. However, in some other cancer types, the rate of ICI response is much lower. For example, in an analysis of anti-PD-1 and anti-PD-L1 therapies in 27 cancer types (8), only 4 cancer types had an objective response rate (ORR) higher than 30%, while 10 of them had an ORR lower than 10%. This includes common cancers such as breast cancer and prostate cancer. Therefore, only a small proportion of patients have benefited from ICI. Furthermore, among people having initial ICI response, a portion of them ultimately developed drug-resistant progressive disease (9).

The highly variable response to ICI across cancer types and cancer patients is due to a multitude of factors. An effective ICI treatment requires priming and activation of a potent cytotoxic T lymphocyte (CTL) response, which is dependent on proper recognition of tumor antigens in an immunogenic micro-environment. In turn, the immunogenic micro-environment needs to permit proliferation of T cells and their infiltration into the tumor (10). Defects in any components of this complex process, intrinsically or developed over time, could affect ICI efficacy in the form of primary, adaptive, or acquired resistance (11). It would thus be very useful to have biomarkers that can predict ICI efficacy, which not only help stratify patients for suitable therapeutic options, but also provide information about resistance mechanisms that may be utilized to identify drug combinations for more successful ICI treatment (12).

A large variety of biomarkers have been suggested to correlate positively or negatively with ICI efficacy (9,13), many of which are functionally related to the mechanistic components of ICI described above. For example, early studies focused on the overall tumor mutation burden, usually quantified by the number of non-synonymous somatic mutations, as a positive biomarker of ICI efficacy. Conceptually, a high mutation burden could produce more neoantigens that can be processed and presented for T cell recognition. Thus, when immune activity is re-established post-blockade, there is greater immune presence in the tumor. This has been supported by clinical and experimental evidence, which shows that a high burden of clonal neoantigens is associated with improved patient survival, increased tumor-infiltrating lymphocytes, and durable ICI response in multiple cancers (14,15). Furthermore, since the immunogenicity of the processed tumor antigens depends on the interaction between the T-cell receptor (TCR) and the peptide–MHC loading complex, the diversity of HLA alleles and the richness and clonality of TCR repertoire have also been proposed as positive biomarkers of ICI response. For example, an early study has found that T-cell population clonality significantly correlates with higher anti-PD-1 efficacy in metastatic melanoma (16). Recently, more studies have also revealed the importance of epigenetic reprogramming as indicators of ICI efficacy (17). In general, the number of ICI-related biomarkers is rapidly accumulating from clinical and pre-clinical studies.

Moreover, in addition to simple biomarkers that are used individually (number of non-synonymous somatic mutations, diversity of the T cell repertoire, etc.), recent studies have also explored more complex computational models that capture intricate interactions among different types of information for predicting ICI efficacy (15,18,19). The latest research makes use of advanced machine learning methods such as those that involve deep artificial neural networks with many layers of connections to capture subtle higher-order features (20).

In general, recent years have seen an explosion of new publications that describe these biomarkers and prediction models (both to be referred to as ‘predictors’ hereafter for simplicity; see explanations below) for ICI efficacy due to the rapidly increasing number of relevant studies. For instance, in 2022 there were more than 5600 clinical trials for seven marketed first-generation ICIs (21). It is extremely difficult to keep track of all new findings about the predictors proposed in the literature. Reading each individual publication in detail is practically infeasible for most clinicians and ICI scientists. Review articles provide crucial summaries of the latest findings, but the time delay caused by the manuscript preparation-review-revision cycle(s) means that by the time a review article is published, a lot of new findings not covered by the review have already been published. Besides, due to length limitations, review articles cannot explain all the technical details of the studies, while details can be critical for the predictors to be effective and for the published results to be reproducible (22–24).

Due to the reasons above, we have developed a knowledgebase about ICI efficacy and a corresponding online portal for easy access to the information stored in our knowledgebase. There are some existing online ICI resources that provide information about and/or implementation of either only a selected small subset of ICI efficacy predictors (25,26), only lists of checkpoint targets and modulators without patient response (27), or multi-omics features associated with ICI responsiveness in only specific cancer types (28). There are also resources that catalog gene expression datasets related to immunotherapy, but not predictors (29). Thus, there is not an existing resource about all types of ICI efficacy predictors, across all cancers. In the current work, we not only comprehensively catalog such predictors in the literature, but also systematically organize the information in a knowledgebase such that users can easily obtain method and result details, and compare across studies with minimal effort.

The primary users of our resource are (i) biomedical scientists and clinicians who want to find existing ICI efficacy predictors for particular types of cancer and ICI drugs of interest, such that they can study the mechanisms of action of the predictors and develop clinical applications of them, (ii) bioinformaticians who want to understand the technical details of specific predictors, such that they can implement them and/or apply them to certain data sets and (iii) data scientists who want to collect information about predictors and data sets, such that they can perform benchmarking studies and develop new predictors with better performance.

## MATERIALS AND METHODS

### Collecting publications related to ICI efficacy

We used a semi-automatic approach to identify publications related to ICI efficacy. We initially performed manual, keyword-based searches on PubMed. In a recent meta-analysis that compared predictors of ICI efficacy (15), the authors provided 20 sets of search terms used to find the 55 predictors included in their comparisons, such as (‘Predictive biomarker’ AND ‘immunotherapy’). We used these search terms to perform our first round of literature searches.

While these initial keyword-based searches did help us obtain publications related to ICI efficacy, testing different combinations of keywords on the user interface of PubMed was tedious due to difficulties in comparing and quantifying results from different searches. We therefore made use of an automatic tool, Literature Scanner (LISC) (30). LISC searches from PubMed using the Entrez Programming Utilities (EUtils) application programming interface (API) and looks for keywords within publication titles and abstracts. Additionally, each LISC search (called a ‘scan’) can be saved in a customizable database structure, which provides a clear record of the searches we have performed and the publications we have identified in all previous searches.

To use LISC, we needed to supply a query in the form of a logical expression of search terms. Because each LISC scan takes one query, we wanted to optimize our query to capture many relevant publications that involve a diverse set of keywords. To do that, we examined the search terms used by the authors of the meta-analysis (15). After removing stop words and combining synonyms, the most common words in their search terms were ‘blockade’, ‘checkpoint’, ‘immunotherapy’ and ‘predict’. For this reason, in the first LISC scan, we used the query ("ICI" OR "Immune checkpoint blockade" OR "Immune checkpoint immunotherapy") AND "predict". While the results contained many publications relevant to ICI efficacy, we found that quite a lot of them involved cancer patients who did not actually undergo ICI treatment (mostly from The Cancer Genome Atlas (TCGA) (31) or the Chinese Glioma Genome Atlas (CGGA) (32)). These publications used established predictors (mostly TIDE (25) and Immunophenoscore (33)) as proxies of ICI response instead of taking actual patient response. We therefore expanded our LISC query to exclude these publications. We also found that some publications used the expression levels of ICI targets (e.g. CTLA-4 mRNA level) as a proxy of ICI response rather than measuring the patient/model organism responses. We found that adding actual ICI drug names to our LISC query effectively reduced the proportion of such publications, probably because publications that mention specific ICI drug names are more likely related to clinical trials of the drugs. Combining all these findings, our final LISC query was ("Pembrolizumab" OR "Tremelimumab" OR "Ipilimumab" OR "Durvalumab" OR "Nivolumab") AND ("Predict" OR "predict response") NOT ("TIDE" OR "immunophenoscore" OR "TCGA"), which effectively retrieved a large number of publications with a high proportion of which being relevant to ICI efficacy predictors.

### Extracting and organizing information from the collected publications

We manually read the collected publications and extracted up to three categories of information from each publication: Information related to the publications themselves (e.g. the journal and publishing year), and information related to the predictors and/or datasets, if available.

The first category of information is general information reported in the publications, including species from which data were obtained, cancer types considered, ICIs administered, and citation details of the publications. Different writing styles and detail across publications result in numerous forms of output for information. For example, one publication may simply define the cancer type of their cohort as ‘melanoma’, while another may add that the cohort is ‘stage 3 melanoma’. This necessitates a system for standardization, such that information collection is unified across publications but at the same time information which the authors potentially found important to mention is retained.

In order to standardize cancer type collection and organization, we defined a multi-level system that allows for multiple levels of details. The first level describes primary sites of cancer initiation, as described in the original publication. This can be at the level of tissue (e.g. urothelium), organ (e.g. liver), or organ system (e.g. hepatobiliary). The second level describes cancer sub-types, such as adenocarcinoma, non-small cell lung cancer, small cell lung cancer, and squamous cell carcinoma in the case of lung cancer. The third level records additional details, such as the stage and grade of the tumors in the study cohort. It is possible for a study to involve multiple types of cancers, in which case we recorded all the relevant information. For studies aimed at testing some very general predictors across many cancer types, we additionally recorded a ‘pan-cancer’ cancer type.

Our approach is different from the approaches used in related studies to extract information from existing literature. For example, Checkpoint Therapeutic Target Database (CKTTD) (27) is an online tool that also involves a manual curation process, providing users with potential ICI druggable targets. A page for a druggable target includes a ‘Tumor Types’ section, which describes the cancer type from the relevant publication. Examples of tumor types include ‘Melanoma’ and ‘Sarcoma’. Given that Melanoma and Sarcoma are from two different cancer classifications (primary site where the cancer first develops vs. histological site where the cancer originates), we found it useful to have our described approach, so that users would be able to confidently find/filter predictors based on their cancer types of interest, without the risk of missing predictors labeled through another classification system. Additionally, we chose primary sites because they are more specific, but our hierarchical approach allows for a histological labelling, if necessary. For example, ‘Tissue – Soft Tissue Sarcoma’ is one such annotation. In this way, we developed a primary site-based system that allows publications to be annotated in a uniform fashion across our knowledgebase. Our organized approach allows users to be able to perform efficient and accurate searching/filtering in our knowledgebase.

Similarly, for the ICIs administered, we defined a multi-level organization of information, with the first level recording the checkpoint molecule (e.g. anti-PD1), the second level recording the exact antibody used (e.g. Nivolumab), and the third level recording additional details (e.g. dosage). Again, it is possible for a study to involve multiple ICIs as well as a combination of them (e.g. anti-CTLA-4 + anti-PD-1 - Ipilimumab + Nivolumab), in which case we recorded all the information.

When some information was not available from the publications, such as cancer sub-types, we permitted the corresponding data entries to contain missing values.

The second category of information we extracted from the publications was about the proposed biomarkers/prediction models of ICI efficacy. We considered a biomarker to be a relatively simple quantity, such as the expression level of a gene, while a prediction model to be a mathematical/computational function. In this sense, a biomarker is a special case of a prediction model with a very simple function. Therefore, we refer to both as a ‘predictor’ in general.

For each predictor, we produced a succinct digest of the method details by summarizing the descriptions provided in the publications, which can come from the main text or supplementary materials. There are many different types of predictors proposed in the literature to be indicative of ICI efficacy. We used a general ‘attribute-value’ approach to record method details, where an attribute defines an aspect of a prediction model (displayed as a section header on the web portal) and its value records the corresponding details. For example, a methodology commonly used in different studies is to perform differential expression analysis between ICI responders and non-responders, followed by an integration of the top genes using a weighted sum or more complex machine-learning methods (34–36). For these types of methods, we created a ‘feature construction’ attribute, which in this example contains information about how useful features are constructed from the raw gene expression data, i.e. the differential expression analysis. By defining a standard set of the most commonly used attributes, method descriptions are recorded systematically and are easier to read. At the same time, new attributes can be created for novel aspects of method descriptions in newly analyzed publications, thereby making our knowledgebase easily extensible.

Given that our knowledgebase is an ongoing, long-term project collecting a wide spectrum of predictors ranging from simple biomarkers to computation-heavy models, there is innate variability of the publications from which the predictors are derived. This is seen in differing writing styles, computational complexity, and detail specificity. Furthermore, predictors can come from experimental, clinical, or computational publications, where different details and nuances are emphasized. Even within computational papers, there can be variation in detail, regarding examples such as model construction (i.e. stating the exact parameters of a specific model), the specific training/testing cohorts (i.e. stating the exact number of patients taken from an external cohort), and sometimes the specific inhibitor name (i.e. solely stating ‘PD-L1’). We find that our ‘attribute-value’ scheme makes it possible to standardize the way information is extracted from each publication by looking for important highlights that would annotate the biological context and discovery/construction of a predictor, while also allotting ‘personalized’ flexibility to each predictor, where unique information is able to be recorded, if applicable. This is again different from CKTTD; while both websites have set fields that define information displayed on the web page (i.e. ‘Definition’, ‘Cohort Details’, and ‘Results’ in the ICI Efficacy Information Portal and ‘Tumor Types’, ‘Function’, and ‘Score’ in CKTTD), our portal maintains flexibility through the ‘attribute-value’ scheme within each field. The ‘Definition’ field's potential to have different subsections that give context to a predictor (i.e. ‘Dataset(s)’, ‘Feature Processing’, ‘Model Processing’), differentiates our knowledgebase from many existing tools, as the methodology is set to preserve the biological context of a predictor for the user. Additionally, tools such as CKTTD or MirGeneDB 2.0 (37) often are databases for biological structures (proteins and microRNA, respectively), and do not require flexible headers for different pages, as the characteristics of a peptide or RNA should not change depending on the publication. However, being that a predictor is curated, the approach of our tool must change.

In addition to method details, we also recorded three pieces of information integral to interpreting and comparing publications’ results. Two mandatory attributes are the target outcome (e.g. RECIST v1.1 (38)) and the direction of correlation of the predictor output with ICI efficacy. The third holds information about each cohort's size and cancer types. Specifically, the size of the biomarker-discovery/model-training cohort(s) and the size of the validation cohort(s) are appended to a ‘Cohort Details’ section visible on every predictor page. The section includes names/references of the cohort(s), the number of patients/samples each cohort contains, the cancer types involved in the cohort, and the figures that display data generated from that cohort, if applicable. Additionally, if the predictor uses data spanning multiple ICI drugs, each cohort's ICI is annotated. When relevant, we also recorded figures and their captions from the results of the publication. For an open-access publication, these figures are shown directly on our web portal; otherwise, hyperlinks to the figures are provided such that users with access right to the figures can view them on the journal web sites by following the hyperlinks.

Across publications, datasets hold integral value for constructing and testing prediction models. While there are studies that directly produce/introduce datasets, many publications rely on obtaining existing datasets from published work. Some of these datasets have high potential to be used for studying ICI efficacy in future research, due to their sample number and data availability. For these datasets, we recorded information about them as the third category of information of our knowledgebase. We defined six mandatory attributes for each dataset, including the number of patients, collection time of patients’ bio-samples (e.g. pre-treatment or on-treatment), number of bio-samples, experimental data obtained from the samples and the corresponding technology (e.g. DNA - Targeted Gene Sequencing), data processing details, and the ICI target. We also recorded some optional attributes when the information was available from the publications, such as bio-sample type (e.g. FFPE derived from tumor or frozen PBMC derived from paired non-cancer samples), clinical information details, and accession numbers of dataset repositories (e.g. dbGaP: phs000452.v2.p1 (39)).

### Knowledgebase design

Since the information in our knowledgebase is highly structured, we designed a relational database schema (Supplementary Figure S1) that flexibly handles the multi-level nature of information and the presence of missing values for better data integrity and consistency across the three main types of contents (publications, predictors, and datasets). Apart from the three main types of contents, some informative attributes mentioned earlier were designed as entities to improve lookup/retrieval and statistical analysis performance. Additionally, we defined registered user and subscriber both as entities for notification preference management and access right control.

For the system design, we used a client-server architecture (Supplementary Figure S2). In the backend, we implemented the database using MariaDB (Community Server Version 10.6, https://www.mariadb.com/), an open-source relational database management system. It supports a large number of concurrent connections without much performance degradation. Data replication can be performed to ensure scalability and high availability. MariaDB provides convenient administration functions and it also works well communicating with our backend server (based on the open-source Python-based Django framework Version 3.2, https://www.djangoproject.com/) to provide a Django admin site for us to easily update and manage the contents in our knowledgebase. The web-based frontend was implemented using the progressive JavaScript framework Vue.js (Version 3.1, https://www.vuejs.org/). It interacts with the backend server through the API provided by the Django REpresentational State Transfer (REST) framework (https://www.django-rest-framework.org/). In such a way, we decoupled the frontend and backend to reduce the chance of simultaneous breakdown of them. This design also makes development easier since debugging can start by determining whether an observed issue is due to the frontend or backend. In addition, upgrade efficiency and scalability of the system are also guaranteed with two set of regulations and resources. The detailed design of the web-based frontend will be introduced in the Results section.

As for security measures, in our database, user email addresses are protected with Advanced Encryption Standard (AES) and user account passwords are protected by the PBKDF2 algorithm. On our website, we used token-based authentication for user identity verification to protect user account information from Cross Site Request Forgery (CSRF) attacks. We also adopted AES to secure communications between our web frontend and backend server through the Django REST API. Finally, we deployed Cloudflare services (https://www.cloudflare.com/website-terms/) to speed up and further strengthen the security of our website connections, with functions such as Content Delivery Network (CDN), Distributed Denial-of-Service (DDoS) protection, and Secure Sockets Layer (SSL)/Transport Layer Security (TLS) encryption.

## RESULTS

### Knowledgebase statistics

Using LISC, we obtained 200 publications from each scan and 416 unique publications in total from three scans. By manually checking 30 random publications of the 200 publications from each scan, we found that the LISC scans achieved a true positive rate of 80%, i.e. 80% of the publications obtained from these scans were truly relevant to ICI efficacy predictors, datasets, or reviews, based on cohorts undergoing ICI. The 20% false positives mainly contained editorials, case reports, publications about predicting cost-effectiveness of drugs, and biomarker proposals from publications without cohorts undergoing ICI.

From these relevant publications, we deposited information about 60 publications, 62 prediction models, and 13 data sets into our knowledgebase. These publications cover 31 cancer types and 46 of their sub-types in total (Table 1). In these publications, the most studied cancer types are respectively skin, lung, and kidney cancers, which are among the cancer types that have had high response rates with ICI (8).

**Table 1.** Cancer types studied in the collected publications

Primary site	Sub-type	Number of publications
Adrenal Gland	Adrenocortical Carcinoma	1
Ampulla of Vater	Ampullary Carcinoma	1
Anal and Anal Canal	(Without sub-type information)	2
Bile Duct	(Without sub-type information)	1
Bladder	(Without sub-type information)	10
Bladder	Transitional Cell Carcinoma	4
Blood	Acute Myeloid Leukemia	1
Blood	Hodgkin Lymphoma	1
Blood	Non-Hodgkin Lymphoma	1
Bone	Sarcoma	3
Brain	Glioblastoma	6
Brain	Glioma	5
Breast	(Without sub-type information)	5
Breast	Breast Invasive Carcinoma	1
Breast	Triple-Negative Breast Cancer	1
Cervix	Endocervical Adenocarcinoma	1
Cervix	Squamous Cell Carcinoma	1
Colorectal*	(Without sub-type information)	7
Colorectal*	Colorectal Adenocarcinoma	1
Embryo	Embryonal Tumor	1
Esophagus	(Without sub-type information)	5
Esophagogastric*	(Without sub-type information)	1
Gastrointestinal*	Neuroendocrine Tumor	1
Gastrointestinal*	Stromal Tumor	1
Head and Neck*	(Without sub-type information)	6
Head and Neck*	Head and Neck Squamous Cell Carcinoma	4
Hepatobiliary*	(Without sub-type information)	2
Kidney	(Without sub-type information)	5
Kidney	Advanced Renal Cell Carcinoma	1
Kidney	Clear Cell Renal Cell Carcinoma	5
Kidney	Kidney Chromophobe	1
Kidney	Kidney Renal Papillary Cell Carcinoma	1
Kidney	Renal Cell Carcinoma	2
Liver	Advanced Hepatocellular Carcinoma	1
Liver	Hepatocellular Carcinoma	4
Liver	Unresectable Hepatocellular Carcinoma	1
Lung	Adenocarcinoma	2
Lung	Non-small Cell Lung Cancer	21
Lung	Small Cell Lung Cancer	2
Lung	Squamous Cell Carcinoma	1
Ovary	(Without sub-type information)	3
Ovary	Ovarian Serous Cystadenocarcinoma	1
Ovary	Sex Cord Stromal Tumor	1
Pan-cancer	(Without sub-type information)	16
Pancreas	Adenocarcinoma	1
Pancreas	(Without sub-type information)	3
Prostate	Adenocarcinoma	2
Salivary Gland	(Without sub-type information)	1
Skin	Cutaneous Carcinoma	1
Skin	Melanoma	31
Skin	Merkel Cell Carcinoma	1
Skin	Non-melanoma	3
Stomach	Adenocarcinoma	1
Stomach	Gastric Cancer	6
Thyroid	Carcinoma	2
Tissue*	Mesothelioma	2
Tissue*	Soft Tissue Sarcoma	1
Urothelium*	Urothelial Cancer	2
Uterus	Endometrial	1
Uterus	Uterine Corpus Endometrial Carcinoma	1

*: Non-organ level primary sites

The ICIs administered in these studies include anti-CTLA-4 (Ipilimumab and Tremelimumab), anti-PD-1 (Camrelizumab, Cemiplimab, Nivolumab, Pembrolizumab, Sintilimab and Toripalimab), anti-PD-L1 (Atezolizumab, Avelumab and Durvalumab), and their combinations.

The predictors came from several major categories, including simple biomarkers, linear combinations of features (such as gene signatures), and machine learning models. Some of the categories contain a number of sub-categories. For example, a variety of machine learning models have been used in constructing the predictors, and we recorded each major model type (support vector machine, random forest, deep neural network, etc.) as a sub-category (Table 2).

**Table 2.** Model types of the predictors proposed in the collected publications

Model type	Sub-type	Number of predictors
N/A		2
Arithmetic Calculation with Biomarkers		21
Arithmetic Calculation with Biomarkers	Copy Number Alterations	1
Arithmetic Calculation with Biomarkers	Gene Expression Signatures	9
Arithmetic Calculation with Biomarkers	Gene Set Enrichment	1
Arithmetic Calculation with Biomarkers	Methylation Status	1
Biomarker	Gene Expression Signature	4
Biomarker	Gene Mutational Signature	6
Biomarker	Genotype Assessment	1
Biomarker	mRNA Expression Level	3
Biomarker	Protein Expression Score	1
Biomarker	Relative Abundance	1
Biomarker	Tumor-infiltrating Lymphocytes (TILs)	2
Correlation Analysis	Gene Expression Correlations	1
Functional Analysis	GSVA	1
Machine Learning	Binary Classifier	1
Machine Learning	DNN	1
Machine Learning	KNN	1
Machine Learning	Logistic Regression	3
Machine Learning	Multiple Kernel Learning	1
Machine Learning	Naive Bayes	1
Machine Learning	PCA	2
Machine Learning	Random Forest	3
Machine Learning	Random Survival Forest	1
Machine Learning	Single Layer Neural Network	1
Machine Learning	SVM	3
Machine Learning	XGBoost	1
Regression Analysis	Linear Regression	1
Regression Analysis	Random Effects Model	1

The predictors required various types of raw data or processed features as input (Table 3), including clinical features (body mass index, smoker status, tumor stage, etc.), genetic/genomic, transcriptomic, epigenomic and proteomic data, information about the microbiome, and imaging features.

**Table 3.** Vocabularies used to define input data required for a predictor

Input data kind	Technology/detail	Number of predictors
Cell	Flow Cytometry	4
Cell	Lymphocytes	2
Cell	Neutrophils	2
Cell	Platelets	1
Clinical	Age	1
Clinical	Antibiotics Taken	1
Clinical	BMI	1
Clinical	Cancer Type	1
Clinical	Chemotherapy Received?	1
Clinical	HGB	2
Clinical	List of Metastatic Organs	2
Clinical	Performance Status	1
Clinical	Sex	1
Clinical	Smoker Status	1
Clinical	Tumor stage	1
Epigenetic	Microarray DNA Methylation	1
Genetic	Somatic Mutations	1
Genetic	Ultra Deep Sequencing	1
Genetic	Whole Exome Sequencing	15
Imaging	Brain MRI	1
Metagenomic	ELISA Test	1
Metagenomic	Shotgun Sequencing	1
Protein	Albumin	1
Protein Level	AFP levels	2
Protein Level	Chemiluminescent Microparticle Immunoassay	1
Protein Level	CyTOF	1
Protein Level	ELISA	1
Protein Level	Immunohistochemical Staining	7
Protein Level	Immunoprecipitation	1
Transcript Level	Microarray Gene Expression	2
Transcript Level	nCounter	5
Transcript Level	qPCR	1
Transcript Level	RNA-seq	26
Transcript Level	RT-qPCR	1
Transcript Level	scRNA-seq	2

The datasets collected were produced from human and mouse samples. Relative to the time of ICI treatment, some of the samples were collected before, during, and after treatment. Clinical outcomes of ICI were defined based on tumor size change (complete response, partial response, stable disease, and progressive disease according to RECIST), progression-free survival, and overall survival. Information recording the total cohort size, types of data (RNA-seq, whole-exome sequencing, etc.) and number of samples for each data type is annotated within the ‘Cohort Size’, ‘Biosample Type’ and ‘Biosample Size’ sections, respectively. Access links are provided so that users can be directly relocated to the data download sites. These websites typically include the Gene Expression Omnibus (GEO) (40), dbGaP (41), cBioPortal (42) and GitHub.

### Web portal functions

Our ICI Efficacy Information Portal is available at https://iciefficacy.org/. It has been running in production mode since July 2022 and used by over 500 unique users. Contents in our database can be accessed through our web portal without logging in. We also provide a free subscription option for users to receive updates from us. Currently, the REST API is not open for public access because we include information of publications that require access rights. We plan to explore the possibility to allow registered users to use the API in future version updates.

**Figure 1.**
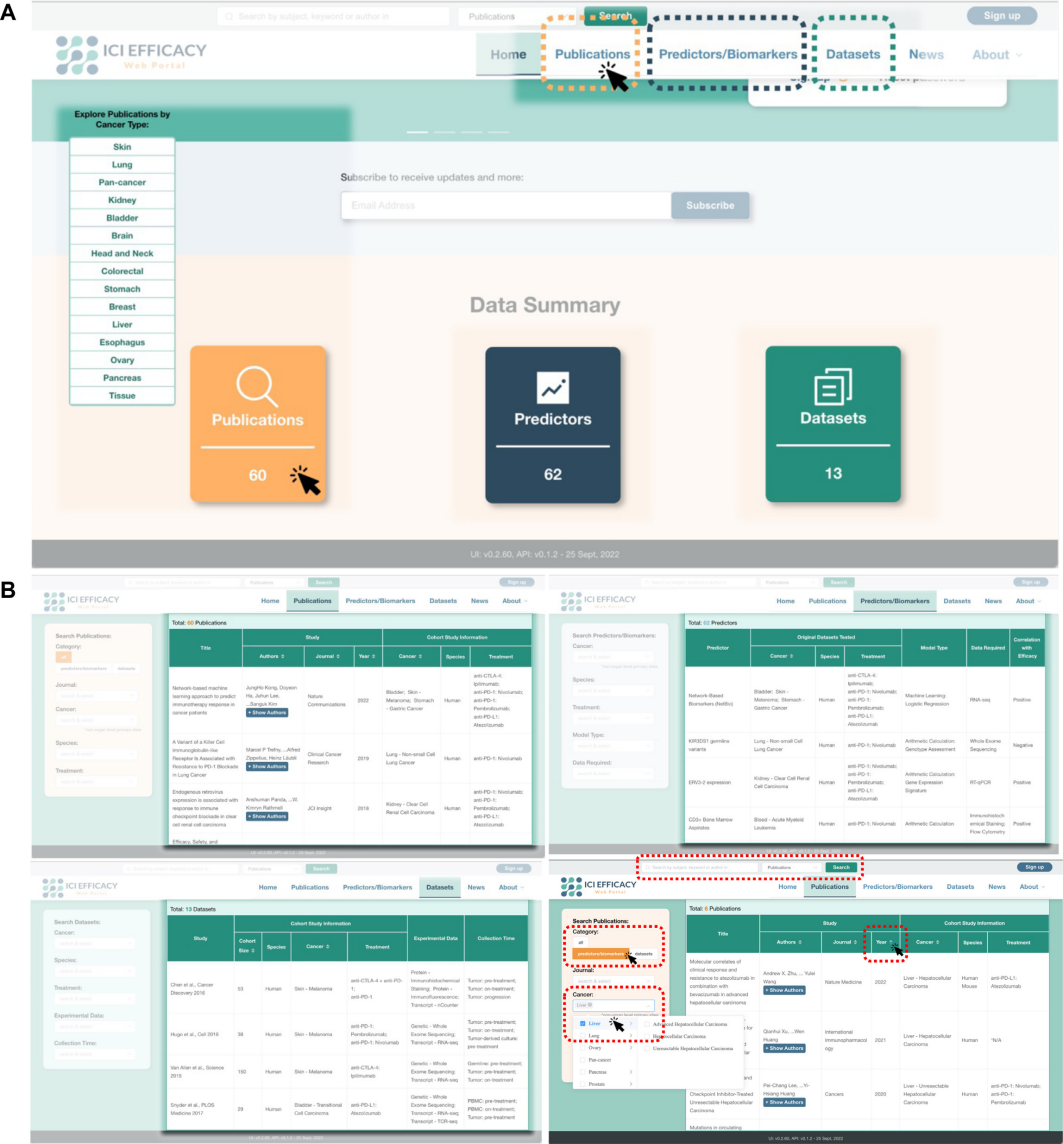
Display of information on our web portal. (**A**) Data summary and entry points of the three main sections, for publications, predictors and datasets, respectively. (**B**) Overview lists of publications (upper left), predictors (upper right), and datasets (lower left), and the filter function (lower right). In the filtering example, only publications that propose a predictor for liver cancers are shown, in reverse chronological order of publication year.

Our web portal is mainly organized into three sections, respectively for displaying information about the publications (https://www.iciefficacy.org/publications), predictors (https://www.iciefficacy.org/predictors), and datasets (https://www.iciefficacy.org/datasets) (Figure 1A). In each section, an overview of the collected information is displayed in a table (Figure 1B). Contents in these tables can be sorted in particular orders by clicking the buttons in the column headers (Figure 1B). The three sections are connected by hyperlinks, to provide quick access to the publications that describe specific predictors and datasets, and vice versa, and the datasets used to discover/train and validate specific predictors.

We also provide three functions for users to perform filtering of contents such that they can efficiently obtain information about entries of their interest. First, on the front page, the cancer types with the largest number of publications in the knowledgebase are listed (Figure 1A). Clicking a cancer type will lead to the list of all publications in which the cancer type was studied. Second, on each of the three main content pages, users can define specific content filters using the control boxes on the left (Figure 1B) such that only entries matching the defined criteria will be displayed. The criteria can be selected from the multi-level, multi-keyword content boxes or typed in manually. Third, free-text search function is provided on both the front page and the three main content pages (Figure 1A, B), which allows users to search for publications, predictors, and datasets that contain the words entered in the title or author list, and in the case of predictors, also in the predictor name, method descriptions and related genes, and in the case of datasets, also in the data processing details.

When a specific entry on the publication list is selected, additional information is provided in a popup window (Supplementary Figure S3A). When a specific entry on the predictor or dataset list is selected, a whole new page is shown to provide additional details (Supplementary Figure S3B, C).

### Case studies

Below we discuss three examples of predictors with varying degrees of complexity and one example of a dataset in our knowledgebase.

### Predictor case study 1: EaSIeR

The first example is Estimate Systems Immune Response (EaSIeR) (19), which is a predictor of high complexity. It first derives five categories of features from bulk RNA-seq data from tumor samples, with 1122 features in total. These features are then used to construct two models, by regularized multi-task linear regression (RMTLR) and Bayesian efficient multiple-kernel learning (BEMKL), respectively. When training these models, instead of using actual patient response as the learning target, 10 transcriptome-based scores of immune response are used as proxy. In the publication, the models were then applied to independent datasets to predict ICI response of patients and evaluate the performance of the models by comparing with actual patient response.

These method descriptions are distributed across the text in the results and methods (titled ‘Experimental procedures’) sections and a figure. Due to the complexity of the methods and the scattered descriptions, it would be difficult to understand the methods without reading the whole publication in detail. The technical depth of the methods may also be difficult for some readers to fully comprehend, limiting the accessibility of this publication.

**Figure 2.**
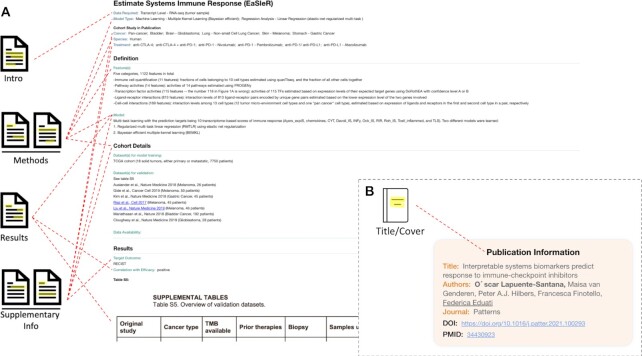
Extraction of information from the publication of the EaSIeR predictor. (**A**) The extraction workflow for the predictor page. Red dashed lines correspond to the publication subsection(s) in which the information is derived. (**B**) The extraction workflow for the citation information page.

In order to break down the publication's complex methods into simplified components, we utilized our ‘attribute-value’ approach explained above. Resulting is a simple digest of the method details (Figure 2A), where the publication's methodology is annotated through a ‘Feature(s)’ section and a ‘Model’ section. The Feature(s) section was further organized by bullet points, explaining each of the five categories of features one by one. Instead of providing all technical details, we focused on explaining the high-level ideas and the corresponding biological concepts. We also used our standard terminology to define the input data required for this predictor, its model type, cancer types of the patients of which this predictor was applied to predict their response, and the ICI treatments these patients received. We also recorded the main testing result of this predictor on the independent cohorts, which are shown in Figure 5 of the original publication. Finally, citation information about this publication was recorded separately (Figure 2B). In this way, we have represented an elaborate computational tool through our schema, such that its methodology, results, and high-level overviews are accessible to biomedical scientists/clinicians, bioinformaticians, and data scientists.

### Predictor case study 2: radiomics-based ML model for glioblastoma

The second example is of medium complexity and is titled ‘Radiomics-based ML Model for Glioblastoma’ (43). Initially, the authors extracted 490 features, including 6 clinical features and 484 imaging features (44,45) from pre-treatment and on-treatment magnetic resonance imaging (MRI). Feature selection then followed, through removing features with high redundancy and low stability. The pre-treatment and on-treatment models had different numbers of remaining features. Finally, the features were integrated using a random survival forest algorithm. The on-treatment model performed much better at predicting overall survival and progression-free survival than the pre-treatment model.

**Figure 3.**
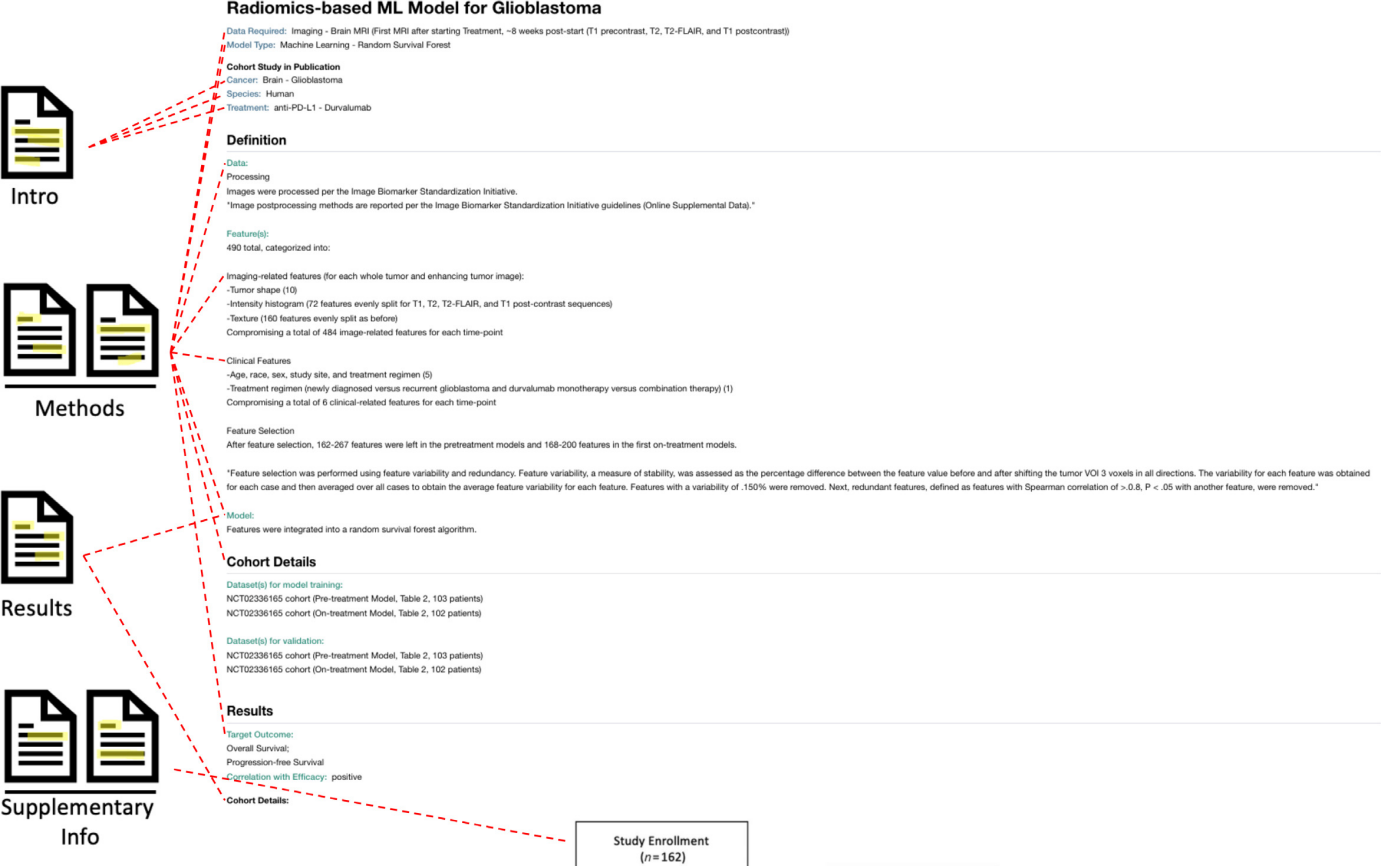
Extraction of information from the publication of the Radiomics-based model for glioblastoma.

As in the previous example, these method descriptions are distributed across the text in the results and methods sections of the paper, though this time, we found the method descriptions in this publication to be relatively straightforward to understand for those without strong computational backgrounds. Thus, we were able to annotate the predictor page for this model as before, once again making use of the ‘attribute-value’ approach (Figure 3). This time, a ‘Data’ section was included in addition to ‘Feature(s)’ and ‘Model’ sections, to list the data processing performed in this example. Additionally, we found this case study to be a good showcase of our ‘(remark)’ field, annotated as (‘Imaging – Brain MRI (First MRI after starting Treatment, ∼8 weeks post-start (T1 precontrast, T2, T2-FLAIR, and T1 postcontrast)’. In this case, the remark captures the nuanced use-case of the radiomics model, in which we excluded the pre-treatment data, because the publication stated that the model did not achieve significant results. While we still find value in recording insignificant biomarkers on our site, by not including it in the data input section, we are making it clearer to users what data they need to successfully utilize the primary model from this publication.

### Predictor case study 3: exosomal PD-L1

**Figure 4.**
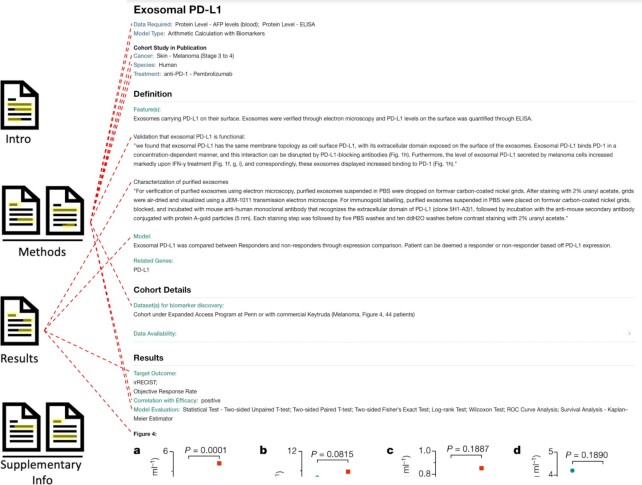
Extraction of information from the publication of the exosomal PD-L1 predictor.

Our third example is of low complexity and is titled ‘Exosomal PD-L1’ (46). It was found that the total expression of PD-L1 from exosome surfaces was positively correlated with efficacy. We have previously mentioned that our usage of the term ‘predictor’ refers to both simple biomarkers and more complex prediction models. The exosomal PD-L1 predictor page is an example of the former. In biomarker manuscripts, there is wide variation in how biomarkers are detected. This variation encompasses different experimental means, such as model systems, organisms and a large array of validation tools (such as microscopy, immunohistochemical staining, and flow-cytometry). For this reason, we have determined that it is more useful for users if we focus on describing the biomarker itself, in addition to the general quantitative approach to correlate it with efficacy, rather than providing extensive details of the experimental procedures (Figure 4). In this way, we are treating biomarkers as predictors on their own, which may then be measured in other data types.

### Dataset case study

**Figure 5.**
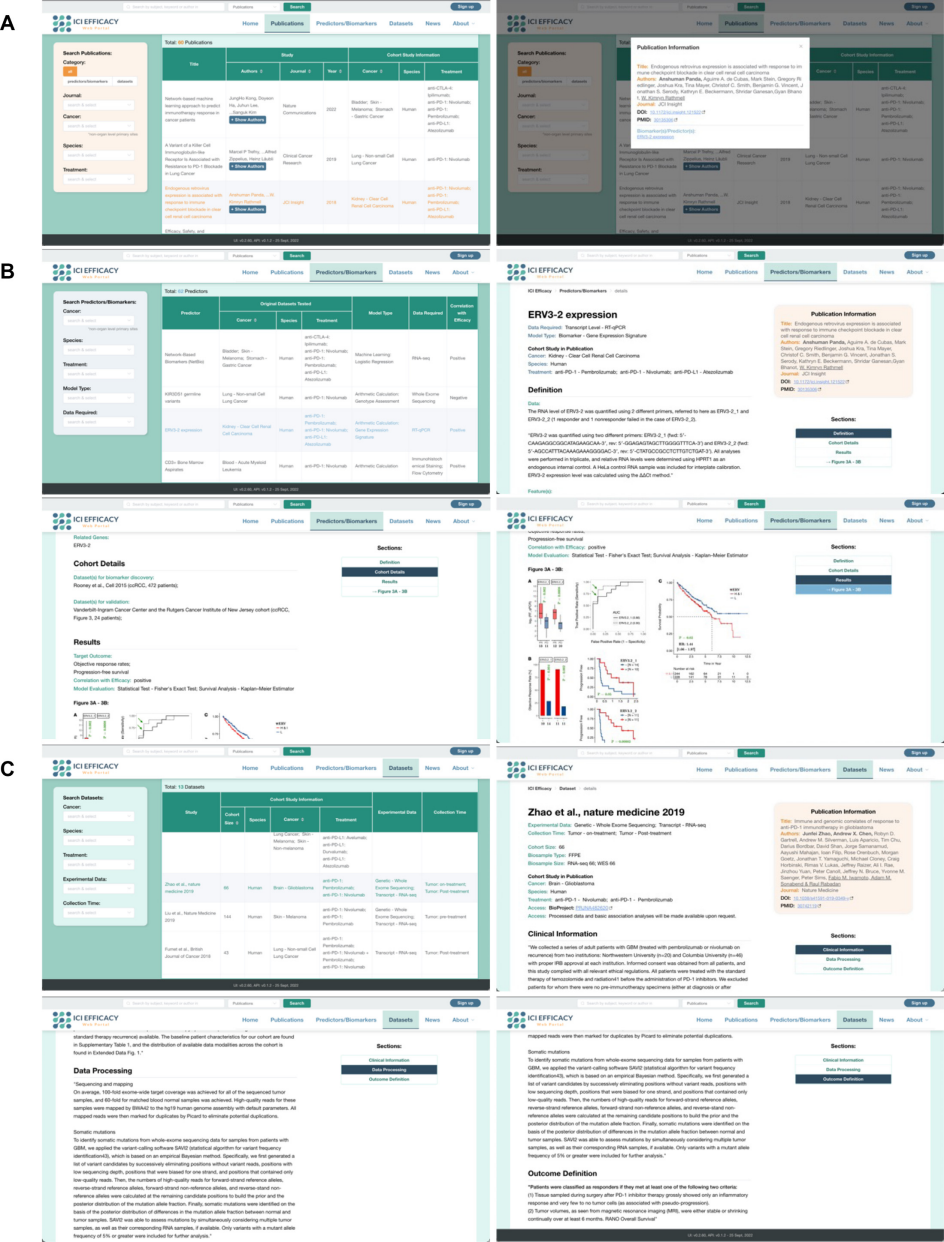
Extraction of information from the publication of the Van Allen *et al.* dataset.

In addition to predictors, our knowledgebase records dataset information. Below is an example of a dataset page for the widely used Van Allen dataset (39) (Figure 5). This dataset includes whole-exome sequencing (WES) and RNA-seq data from 150 patients, made accessible in 2016.

Users exploring annotated datasets immediately see information useful to understand the biosample type, sample size, and associated clinical information the dataset contains. In particular, we used:

- The ‘Experimental Data’ attribute to record the RNA-seq and WES data included in the dataset
- The ‘Collection Time’ attribute to record in detail that both tumor and non-tumor (tumor-adjacent normal tissue) samples were collected pre-treatment, while tumor samples were also obtained during treatment
- The ‘Cohort Size’ attribute to record that there were 150 patients
- The ‘Biosample Type’ attribute to record that the samples were in the form of formalin-fixed, paraffin-embedded (FFPE) tissues
- The ‘Biosample Size’ attribute to record that there were 110 samples with WES data, 42 samples with RNA-seq data, with 40 of them in common
- The ‘Cancer’ attribute to record that the cancer type was skin cancer, with melanoma as the subtype
- The ‘Species’ attribute to record that all data came from human samples
- The ‘Treatment’ attribute to record that the ICI administered was the anti-CTLA4 antibody Ipilimumab
- The ‘Access’ attribute to record that the study accession ID in dbGaP is phs000452, the data set version is 2, and the participant set version is 1 (phs000452.v2.p1)

Text outlining clinical information and data processing summaries are also extracted from publications. This information includes patient inclusion criteria and outlines tools and general methodology required for processing. Finally, the outcome definition lists the metric used to evaluate patient outcome. Importantly, users are directly linked to the site that data can be downloaded, making this a very useful tool for ICI researchers.

Ultimately, the dataset detail page provides users a helpful summary of existing ICI-related datasets.

## DISCUSSION

In this paper, we present our knowledgebase, a novel, comprehensive, and manually curated resource that captures the ever-growing number of ICI datasets and efficacy predictors. We include diverse publication types, ranging from those describing simple experimental biomarkers to complex computational models, organized such that they can be useful to ICI scientists and clinicians, bioinformaticians, and data scientists. Our user-friendly resource presents a powerful opportunity as a new tool for browsing existing publications, as well as developing new insights.

We have described the workings behind our knowledgebase and online portal that led to easy access to information about ICI efficacy. Given the diverse types of cancers ICIs, and predictors considered in the literature, we have defined flexible multi-level schemes to systematically record their corresponding information. To date, 60 publications, predictors, or datasets have already been recorded in our knowledgebase. We will continue adding more content in coming releases.

To scale up our data acquisition effort, we plan to increase the level of automation of our workflows. In terms of discovering ICI efficacy-related publications, we plan to utilize existing tools which specialize in literature discovery. One such tool is Elicit (https://elicit.org/), which uses the Semantic Scholar API to perform initial retrieval of titles and abstracts, followed by a ranking of these results based on their relevance to the user query, using the GPT-3 (Generative Pre-trained Transformer 3) autoregressive language model (47) and a finetuned T5 encoder-decoder model (48). In our preliminary trials of this tool, we obtained ICI-related publications with high precision. Additionally, given that we have 60 publications in our knowledgebase, we plan to perform citation tracing to identify other publications that cite or are cited by these publications.

In terms of extracting information from the publications, we plan to explore artificial neural networks based on the transformer architecture (49), which have been highly successful in natural language processing tasks.

An important use of our resource is constructing new machine learning models that integrate many types of predictors previously proposed. For example, a recent study (15) has surveyed the literature to obtain 55 predictors proposed to be indicative of ICI efficacy. Whole-exome sequencing and transcriptome data were then obtained from previous studies and reprocessed in a uniform way to compute values of these predictors from each patient. After a feature selection procedure, the predictors were further integrated into a gradient boosting tree model. We envision that this type of large-scale integration will become increasingly common in the research of ICI efficacy as more predictors are proposed and more datasets become available. Our resource will make the discovery of the published predictors and datasets very easy. Our structured records of the details will also enable quick initial selection of suitable predictions for integration.

In the long term, it is necessary to conduct large-scale benchmark studies, such that the different predictors can be compared objectively using the same datasets and evaluation procedures. We plan to contribute to this benchmarking effort by providing the implementations of some of the predictors on our web portal, such that users can apply them on their own data. We also plan to validate the proposed predictors with the datasets used across published studies, to evaluate the reproducibility of the reported results.

In addition to efficacy, another important research direction of ICI is immune-related adverse events (irAEs). There are a growing number of studies that identify and characterize predictors of irAEs (9). The methodology and data types involved share some similarity with those for studying ICI efficacy, but also encompass unique aspects, such as the many categories of irAEs, and the corresponding treatments. We hope to extend our knowledgebase and web portal design to cover irAEs in the future, in the same standard yet flexible manner implemented in our knowledgebase.

## DATA AVAILABILITY

No new data were generated or analysed in support of this research.

## Supplementary Material

zcad012_Supplemental_File
