# Supplementary material for: ICI efficacy information portal: a knowledgebase for responder prediction to immune checkpoint inhibitors
Source: NAR Cancer. 2023 Mar 3;5(1):zcad012. doi: 10.1093/narcan/zcad012 (PMC9984987; doi:10.1093/narcan/zcad012)
Supplement: zcad012_Supplemental_File [file zcad012_supplemental_file.docx]

**SUPPLEMENTARY DATA**

**Supplementary Figures**

*
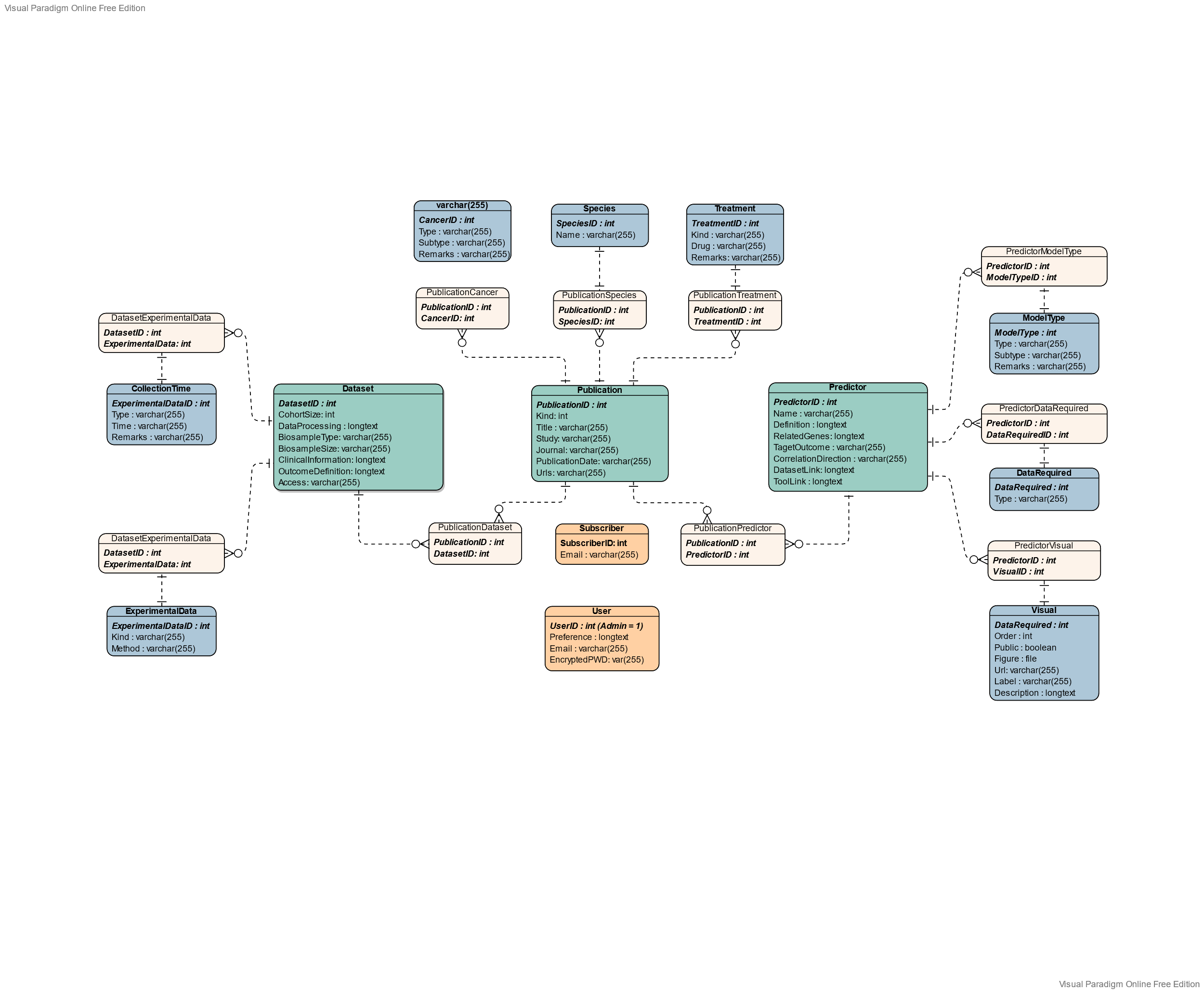
*

Supplementary Figure 1. Schema design of our relational database. The three main entity sets (in green) are respectively for publications, datasets, and predictors Each of them is associated with various attributes to define their properties and multi-level classifications. Datasets and predictors are also linked to their original publications. Our database also contains some entity sets for user management functions.


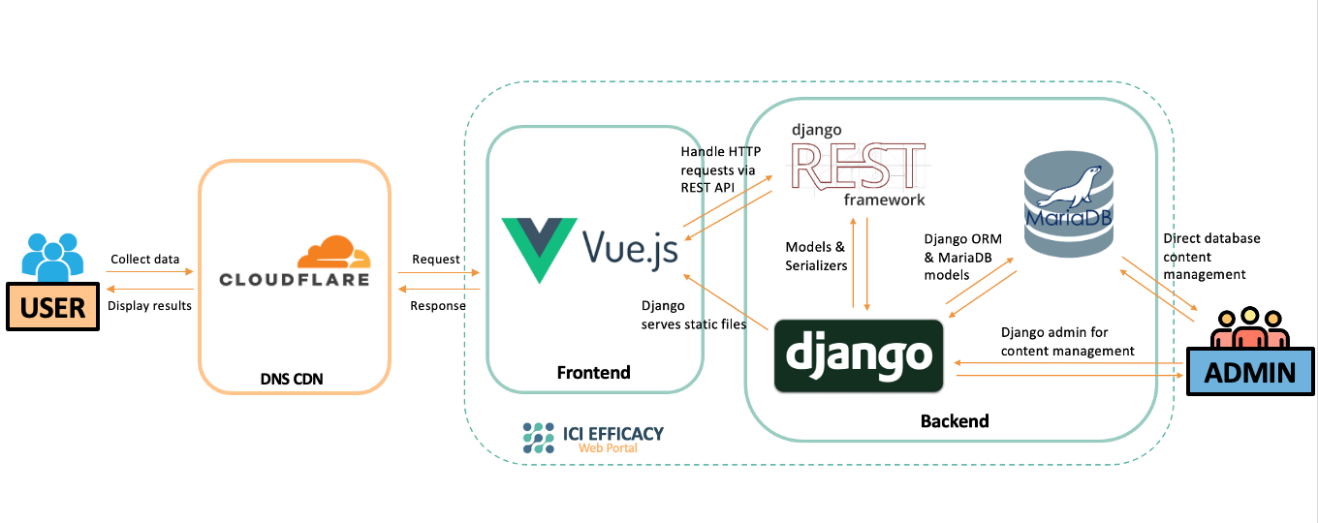


Supplementary Figure 2. The overall system architecture. Users request for specific contents on our web portal through the Cloudflare’s global distributed network and reach our frontend implemented with Vue.js. The frontend communicates with the Django backend server through the API provided by the REST framework. The backend server executes the requests by communicating with the MariaDB database. Then, results are provided back to the frontend in the form of static files and, finally, display to users through the content delivery network. Administrators update contents of the knowledgebase either by using the content management function provided by Django or interacting with the database management system directly.

Supplementary Figure 3. Display of detailed information of individual entries. A) By clicking a specific row in the publication table, a popup window is shown to provide additional information about the publication, with hyperlinks to related predictor(s) and dataset(s). B) By clicking a specific row in the predictor table, a new page is shown to provide additional information about the methods, datasets for predictor discovery/training and validation, and testing results of the predictor. C) By clicking a specific row in the dataset table, a new page is shown to provide additional information about clinical information and processing steps of the dataset.
